# HMGA1 in cancer: Cancer classification by location

**DOI:** 10.1111/jcmm.14082

**Published:** 2019-01-07

**Authors:** Yuhong Wang, Lin Hu, Yushuang Zheng, Lingchuan Guo

**Affiliations:** ^1^ The First Affiliated Hospital of Soochow University Department of Pathology Suzhou Jiangsu China; ^2^ Institutes of Biology and Medical Sciences Soochow University Suzhou Jiangsu China

**Keywords:** cancer, gene function, HMGA1

## Abstract

The high mobility group A1 (HMGA1) gene plays an important role in numerous malignant cancers. HMGA1 is an oncofoetal gene, and we have a certain understanding of the biological function of HMGA1 based on its activities in various neoplasms. As an architectural transcription factor, HMGA1 remodels the chromatin structure and promotes the interaction between transcriptional regulatory proteins and DNA in different cancers. Through analysis of the molecular mechanism of HMGA1 and clinical studies, emerging evidence indicates that HMGA1 promotes the occurrence and metastasis of cancer. Within a similar location or the same genetic background, the function and role of HMGA1 may have certain similarities. In this paper, to characterize HMGA1 comprehensively, research on various types of tumours is discussed to further understanding of the function and mechanism of HMGA1. The findings provide a more reliable basis for classifying HMGA1 function according to the tumour location. In this review, we summarize recent studies related to HMGA1, including its structure and oncogenic properties, its major functions in each cancer, its upstream and downstream regulation associated with the tumourigenesis and metastasis of cancer, and its potential as a biomarker for clinical diagnosis of cancer.

## 1 INTRODUCTION: HMGA1 PROTEINS

High mobility group A (HMGA) proteins are small nuclear proteins with high mobility. The HMGA family consists of four members: three HMGA1 protein isoforms because of alternative splicing, HMGA1a, HMGA1b and HMGA1c, and the fourth member HMGA2. The first three members are located on chromosome 6p21, whereas HMGA2 is transcribed by a separate gene on chromosome 12q15.^1^ The HMGA family lacks intrinsic transcriptional activity, but it can remodel chromatin structures and later regulate the interaction between the transcriptional regulatory proteins and downstream DNA, the so‐called “architectural transcription factors,” each of which contains three N‐terminal motifs, known as an “AT‐hook.” The HMGA family preferentially binds to other special DNAs, which have AT‐rich sequences and recruit the DNAs to HMGA family binding sites. HMGA proteins also have an acidic C‐terminal, which may be important for protein‐protein interactions or for inducing specific proteins to the enhanceosome.^2^

It is reported that the high expression of HMGA1 has an essential role in embryonic development. However, in terminal mature differentiation organization, the HMGA1 protein is not detected or is detected at a very low expression. In 1983, Lund et al first discovered HMGA1 expression in aggressive cervical cancer cells.^3^ Following that discovery, increasing research has provided compelling evidence of elevated HMGA1 expression in malignant cancer,^4^, ^5^ regardless of where the neoplasms originated (Table 1), including in epithelial cancers such as breast cancer,^6^ lung cancer,^7^, ^8^ colorectal cancer^9^, ^10^ and uterine cancer,^11^ and mesenchymal tumours such as lipoma/liposarcoma,^12^ glioma/glioblastoma,^13^ fibroma/fibrosarcoma,^14^ leiomyoma^15^ and osteosarcoma.^16^ Collectively, these studies reveal that HMGA1 has an important role in tumourigenesis and tumour progression and that the expression level of HMGA1 negatively correlates with clinical prognosis.

**Table 1:** The role of HMGA1 in epithelial cancer and in mesenchymal tumours

Tumour type	Clinical significance	Target gene
Cancer originated from epithelial tissue		
Thyroid cancer	P, I, M, DB	S100A13, TGF‐β1, HAND1, p53
Gastric cancer	P, I, M, DB	let7
Liver cancer	CP	
Cholangiocarcinoma	P, T, DR	
Pancreatic cancer	T, DB, DR	COX2, insulin receptor, MMP9, p‐Akt
Ovarian carcinomas	P, M, S, DB, DR	ABCG2
Cervical cancer	I, M, DB, DR	MMP2, HPV E6/E7, COX2
Lung cancer	P, I, T, DB, DR	miR222, miR26a, miR26, PPP2R2A, IL24, IL6, CK2,MMP2, p‐Akt
Breast cancer	P, M, T, S, CP, EMT, promoting DNA repair	miR625, miR26a, miR181b, Let7a, CBX7,BRCA1, KIT ligand,DNA Ligase IV, CCNE2, TGF‐β1
Colorectal cancer	I, S, DB, DR, chromosome instability	GLUT3, β‐catenin, p53, Sox9, miR137, miR138, miR214
Prostate cancer	P, M, DB, CR, androgen independence	miR296, miR195, miR765, Let7b, MMP2, BCAS2, estrogen receptor β
Cancer originated from mesenchymal tissue		
Lipoma/liposarcoma	P, CR	LPP/TPRG1, E2F
Leiomyoma	CR	
Osteosarcoma	P, I, M	miR142‐3p
Hemangioma	CR	TBL1XR1
Medulloblastoma	P, I, M, DB	CRMP1, cdc25A, hsa‐miR124a
Glioma/glioblastoma	P, S, CP, DB, DR, angiogenesis	miR1297, miR296‐5p, HIF1A‐AS2, Sox2
Dermatofibroma & dermatofibrosarcoma	DB	
Angiomyxoma & angiomyofibroblastoma	CR	

P, proliferation; I, invasion; M, metastasis; T, tumourigenesis; S, stemness; DB, diagnostic marker; CP, clinical prognosis; DR, drug resistance; CR, chromosomal rearrangement.

As HMGA1 is overexpressed in embryonic tissues, comprehending the role of HMGA1 in cancer is essential for our understanding of HMGA1‐mediated tumourigenesis. HMGA1 functions as an oncogene through transcriptional regulation and protein‐and‐protein interaction. For example, in breast cancer, the expression of HMGA1 protein level indicates the adverse outcome of clinical prognosis. Zhou et al also found that miR‐625 suppresses cell migration and proliferation by decreasing HMGA1 protein expression.^17^ Overexpression of HMGA1 is correlated with human epidermal growth factor receptor 2 (HER2) and studies show that TGF‐β1 induces HMGA1 expression to promote breast cancer.^18^ Early studies show that HMGA1 research in colorectal cancer focused on overexpression leading to a worse clinical prognosis.^19^ Nearly 5 years of research shows that HMGA1 promotes colorectal cancer development, primarily through transcriptional regulation of such targets as the Wnt signalling pathway,^20^ miR‐137^21^ and miR‐214.^22^ The rise of metabolomics in recent years has also observed that HMGA1 can increase glucose uptake, promote aerobic glycolysis and promote the development of colorectal cancer.^10^, ^23^ Additionally, in HMGA1 transgenic mice, the faecal metabolome can be used as a non‐invasive diagnostic marker of early colorectal cancer.^24^

In this article, we discuss the current findings on HMGA1 in tumours as well as recent progress in characterizing the molecular mechanism and function of HMGA1 in the tumourigenesis and malignant progression of numerous cancers. We hope this review will provide a stronger understanding of this small but important oncogene and will inform future studies aimed towards the development of targeted therapy and biomarkers for clinical diagnosis.

## 2 CLINICAL PROGNOSIS

In all of the literature, HMGA1 is an oncogene in numerous cancers. In this paper, we review cancers classified by location and consider whether HMGA1 serves as a biomarker of clinical prognosis. We systematically classify the expression level and the clinical prognosis of HMGA1 in head and neck cancers, thoracic cancers, abdominal cancers and reproductive system cancers (Table 2). Table 2 shows the specific role of HMGA1 in these cancers.

**Table 2:** The systematic classification of the HMGA1 expression level and clinical prognosis in cancer

Tumour type	Expression level	Clinical prognosis	Ref.
Pituitary tumours	High	Poor	Wang et al (2010)
Glioma/Glioblastoma	High	Poor	Donato et al (2004); Pang et al (2012)
Thyroid cancer	High (specific)		Czyz et al (2004); Kim et al (2000)
Lung cancer	High (specific)	Poor	Zhang et al (2015)
Breast cancer	High	Poor	Sepe et al (2016); Huang et al (2015)
Colorectal cancer	High	Poor	Takahashi et al (2013)
Hepatobiliary cancer	High	Poor	Chang et al (2005)
Pancreatic carcinoma	High (specific)	Poor	Hristov et al (2010)
Prostate cancer	High	Poor	Leman et al (2003)
Ovarian carcinomas	High	Poor	Zhou et al (2015)
Testicular seminomas	High		Chieffi et al (2013)

Specific: in a specific cancer subtype.

In pituitary tumours, Wang et al reported that an increased expression of HMGA1 in pituitary adenomas, whereas in normal tissues it was negative, and HMGA1 was significantly more expressed in invasive adenomas.^25^ In gliomas, Pang et al and Donato et al revealed that HMGA1 expression was increased in gliomas compared with normal adjacent tissue; however, expression was lower than in glioblastomas. HMGA1 expression level was correlated with the histological grade in gliomas and in glioblastomas.^26^, ^27^ A series of studies have shown that HMGA1 expression is increased in thyroid cancer, especially in follicular carcinoma, but is not detected in normal tissue.^28^, ^29^ Zhang et al showed in 2015 that HMGA1 expression was increased in non‐small cell lung cancer (NSCLC), which correlated with clinical prognosis.^30^ In another thoracic cancer, breast cancer, many studies have confirmed increased HMGA1 expression in cancer tissue and its association with poor clinical prognosis.^6^, ^31^ Many articles have reported on HMGA1 in colorectal cancer; for example, Balcerczak et al and Takahashi et al reported an increase in HMGA1 expression in colorectal cancer, especially in small‐sized tumours (<2 cm), and the utility of HMGA1 as an indicator of lymph node metastasis.^19^ Chang et al revealed HMGA1 as a prognostic marker in hepatocellular carcinoma because its expression is higher in carcinoma tissue than in normal tissue, and its high expression predicts poor prognosis.^32^ In pancreatic ductal adenocarcinoma, Hristov et al showed a similar result for the role of HMGA1.^33^ In male reproductive system cancers, Leman et al found evidence that HMGA1 correlates with the clinical prognosis of diagnosed prostate cancer (PCa).^34^ In female reproductive system cancers, a similar outcome was confirmed. Moreover, Zhou et al showed that urine HMGA1 expression can serve as a non‐invasive prospective diagnostic indicator.^35^

Overall, it appears that HMGA1 has an important role in cancer, that HMGA1 expression is increased in cancer, and that elevated HMGA1 expression serves as a predictor of poor clinical prognosis. In the following review, we summarize the particular role of HMGA1 in each cancer type.

## 3 HEAD AND NECK CANCER

**Figure 1:**
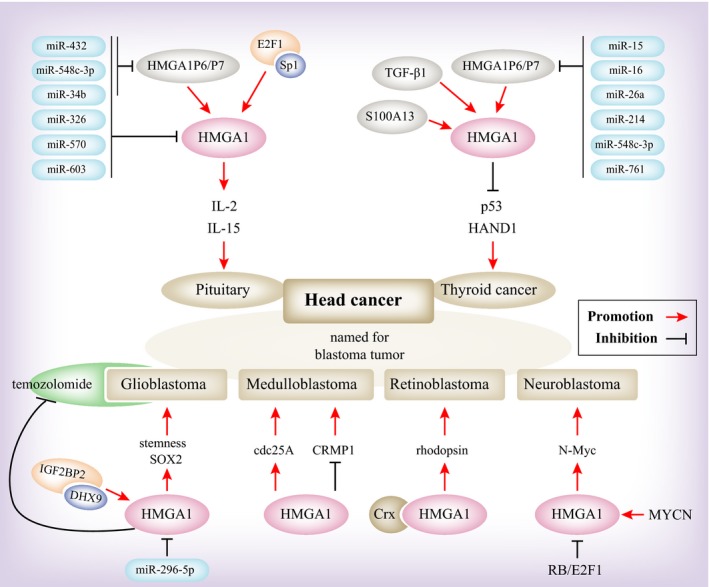
A model depicting the upstream and downstream regulation of HMGA1 in head and neck cancers

According to recent findings, HMGA1 is a potential biomarker for clinical diagnosis in head and neck cancer. Relevant findings primarily focused on pituitary tumours, named for blastoma tumours and thyroid tumours, and we summarize the results for these types of cancer (Figure 1).

In pituitary tumours, HMGA1 transgenic mice tend to develop growth hormone/prolactin cell pituitary adenomas by inducing the expression of IL‐2 and IL‐15 proteins.^36^ The HMGA1 pseudogenes HMGA1P6 and HMGA1P7 act as competitive endogenous decoys for HMGA1 and contribute to pituitary tumourigenesis by increasing the level of HMGA1.^37^ The expression profile of microRNA in the pituitary identified a set of miRNAs that are down‐regulated, such as miR‐34b, miR‐326, miR‐432, miR‐548c‐3p, miR‐570 and miR‐603, which target HMGA1, HMGA2 and E2F1. The high expression of these target genes plays an important role in pituitary tumourigenesis.^38^ These studies show that HMGA1 promotes the progression of pituitary adenomas, but not HMGA1 function. By functional experiments and knockout mouse experiments, it has been observed that Sp1 interacts with E2F1 to promote HMGA1 expression and that deregulation of RB/E2F1 significantly contributes to HMGA1 deregulation in the pituitary.^39^

This group cancer type is named for blastoma tumours, mainly including glioblastomas, medulloblastomas, retinoblastomas and neuroblastoma. For example, in glioblastoma, the expression of HMGA1 is correlated with malignant progression, histological grade, and time to recurrence,^40^ while it is interesting that HMGA1 expression is significantly correlated with glioblastoma stem cells (GSCs).^41^ One potential mechanism is that HMGA1 is regulated by miR296‐5p, but HMGA1 can promote the transcription of SOX2 and thus promote the maintenance of GSCs.^42^ Another article reported that IGF2BP2 and DHX9 bind to each other and then promote HMGA1‐mediated modulation of GSC responses to hypoxic stress.^43^ HMGA1 maintained the GSC regulatory property and led to temozolomide resistance.^13^ In medulloblastomas, it has been reported that HMGA1 can promote the progression of medulloblastoma by promoting cdc25A expression or inhibiting CRMP1 expression.^44^, ^45^ In retinoblastomas, HMGA1 expression is correlated with clinical prognosis^46^ through the same mechanism of RB/E2F1 deregulation by HMGA1.^47^ In neuroblastoma, HMGA1 has been reported as a biomarker for diagnosis and prognosis.^48^ Studies have shown that HMGA1 promotes malignant neuroblastoma by inducing expression of the transcription factor N‐Myc.^49^ Giannini et al revealed that MYCN activated a luciferase reporter expressing the HMGA1 promoter, which contains the first three transcription factor binding sites.^48^

In thyroid cancer, high HMGA1 expression can be used as a diagnostic marker of thyroid follicular cancer to distinguish between nodular thyroid and thyroid cancer.^29^ It was reported that HMGA1 has a vital role in thyroid tissue through inhibition of p53^50^ and HAND1^51^ and induction of TGF‐β1^52^ and S100A13.^53^ The HMGA1P6 and HMGA1P7 also functions in promoting the malignant progression of thyroid cancer.^54^

## 4 THORACIC CANCER

**Figure 2:**
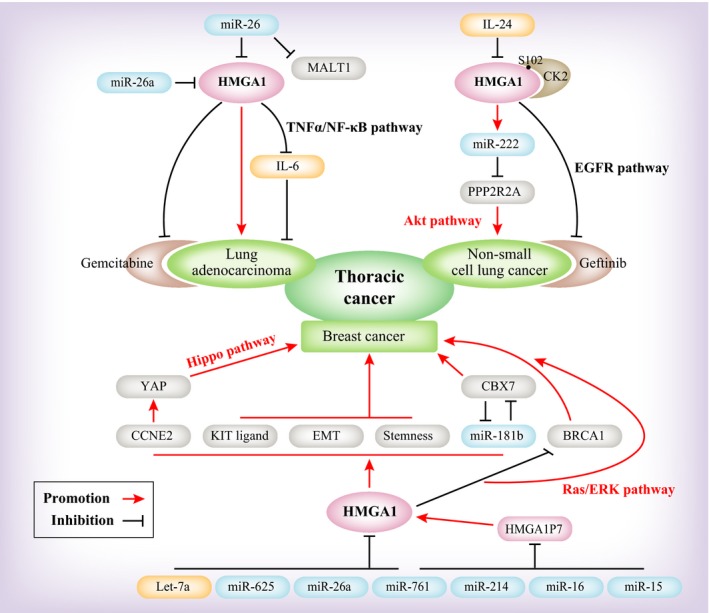
A model depicting the upstream and downstream regulation of HMGA1 in thoracic cancers

In thoracic cancer, the studies focused primarily on lung cancer and breast cancer. These findings confirmed that HMGA1 could be used as a diagnostic and prognostic biomarker. Research on lung cancer primarily concerns NSCLC, while other research is focused on breast cancer. In this section, we expand on the findings for lung cancer and breast cancer (Figure 2).

In NSCLC, using the protein levels or the circulating blood, expression level of HMGA1 as a biomarker for the diagnosis of NSCLC^30^ has been studied. The association of SIRT1 expression with HMGA1 expression can be used as a predictor of the prognosis and progression of NSCLC.^55^ The level of miR‐222 is directly increased by HMGA1 and miR‐222 led to an obvious increase in p‐Akt levels by inhibiting PPP2R2A, thereby promoting the progression of NSCLC.^56^ Based on these experimental results and recent findings from subsequent studies, IL‐24 inhibits HMGA1 and reduces the expression of miR‐222, inhibiting p‐Akt and thereby suppressing NSCLC.^57^ Regarding drug resistance, HMGA1 knockdown or mutation restored the efficacy of gefitinib through the reactivation of EGFR signalling in drug‐resistant NSCLC cells.^58^ Wang et al demonstrated that knockdown of HMGA1 or mutation of S102 of HMGA1, a CK2 phosphorylation site, restored the efficacy of gefitinib through the reactivation of the downstream signalling pathway of EGFR in drug‐resistant NSCLC cells.^58^

Immunohistochemistry and statistical analysis of HMGA1 in breast cancer showed that its expression is correlated with the histological grade, clinical stage, tumour size, lymph node metastasis, distant metastasis and whether it correlates with triple‐negative breast cancer. These findings indicated that HMGA1 expression could be used as a biomarker of breast cancer.^59^ Another paper showed that HMGA1 is transcriptionally activated by the KIT ligand (KL) promoter, which implicates serum KL as a diagnostic marker for HMGA1‐positive carcinomas.^60^ Studies reported down‐regulation of miRNAs in breast cancer, primarily miR‐625, miR‐26a and let‐7a, which inhibit the expression of HMGA1 to suppress breast cancer, thereby elucidating the molecular role of HMGA1 in malignant progression.^17^, ^61^ Additionally, the HMGA1 pseudogene HMGA1P7 can competitively bind to endogenous RNA and thus promote the progression of breast cancer. Mansueto et al confirmed that HMGA1 promotes breast cancer progression by promoting miR‐181b and inhibiting CBX7 expression.^61^ In basal‐like breast cancer and triple‐negative breast cancer, it has been reported that HMGA1 promotes malignant progression and predicts clinical outcome by promoting the transformation of tumour cells into breast stem cells and maintaining stemness.^4^, ^62^ Among these breast cancers, HMGA1 can activate YAP through cyclin E2 (CCNE2), promoting nuclear localization and basal‐like breast cancer progression.^63^ In MCF‐7 breast cancer cells, the data show that HMGA1a increases the activity of the Ras/ERK signalling pathway to promote malignant progression.^64^ Other findings show that TGF‐β1 induces HMGA1 promoter activity during breast cancer progression.^18^ With respect to drug resistance, HMGA1 enhances DNA ligase IV activity and influences DNA repair, thereby reducing the killing effect of chemotherapeutic drugs on tumour cells.^65^ In sporadic breast carcinoma, HMGA1 proteins negatively regulate the BRCA1 gene, which is involved in aggressive mammary carcinomas.^66^

## 5 ABDOMINAL CANCER

**Figure 3:**
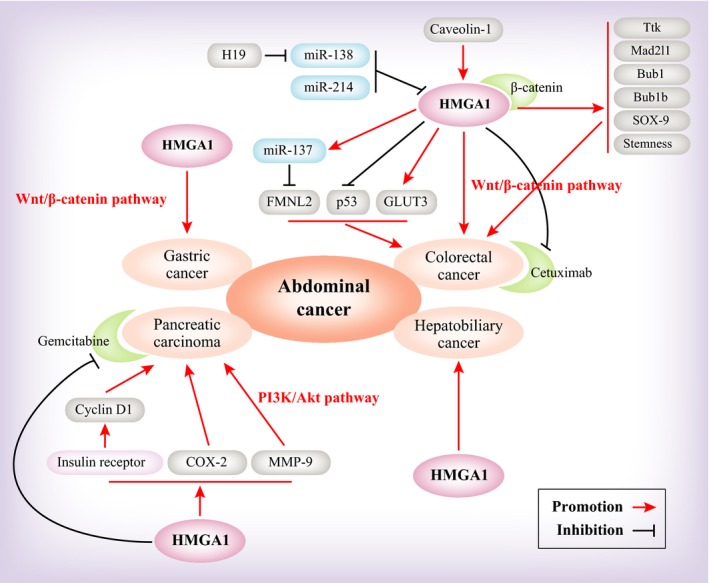
A model depicting the upstream and downstream regulation of HMGA1 in abdominal cancers

In addition to head cancer and thoracic cancer, there is a range of abdominal cancers, including gastric cancer, colorectal cancer, liver cancer, cholangiocarcinoma and pancreatic cancer. In this section, we analyse the findings on each of these abdominal cancers (Figure 3). First, the data confirmed that HMGA1 is an oncogene promoting tumourigenesis in abdominal cancer.

In gastric cancer, there is relatively little research on HMGA1. HMGA1 is associated with the malignant phenotype based on immunohistochemical staining of HMGA1 in gastric cancer tissues and relevant normal tissues.^67^, ^68^ However, the function of HMGA1 is unknown. Only one paper has reported that HMGA1 increased the expression of the Wnt/beta‐catenin pathway. Cytofunction and transgenic mouse experiments confirmed that HMGA1 maintained cell proliferation and was down‐regulated by beta‐catenin or its downstream factor c‐Myc.^69^

In colorectal cancer, HMGA1 protein expression in 81 paired tumour tissues and matched, adjacent, non‐malignant tissues indicated that HMGA1 is associated with the tumours, especially advanced tumours and lymph node metastases.^9^ Another paper showed that in smaller sized (<2 cm) invasive tumours, high HMGA1 expression can increase lymph node metastasis.^19^ The faecal metabolome reveals that the expression of the HMGA1 protein is associated with abnormal proliferation in the Hmga1 transgenic mouse intestinal epithelium. This finding is notable because faecal metabolomic analysis can serve as a non‐invasive screening tool in the early precursor lesions of colorectal cancer.^24^ In the regulation of metabolites, HMGA1 contributes to CRC by inducing fatty acid synthesis and aerobic glycolysis.^10^, ^23^ Above all, we are certain that HMGA1 can be used as a diagnostic indicator of CRC. To determine the mechanism of HMGA1 function, we reviewed the findings from recent years. First, HMGA1 can induce chromosomal instability in CRC by the regulation of spindle assembly checkpoint genes, such as Ttk, Mad2l1, Bub1 and Bub1b.^70^ Second, miRNAs, such as miR‐137, miR‐138 and miR‐214, have important roles in inhibiting the function of HMGA1 and suppressing CRC progression.^21^, ^22^, ^71^ Third, Wnt/β‐catenin signalling is a classical pathway to promote tumourigenesis in colorectal cancer. The data show that HMGA1 can regulate Wnt signalling to promote CRC progression and “build” an intestinal stem cell niche through inducing SOX9, interacting with β‐catenin and specifically binding to the β‐catenin/TCF‐4 complex.^20^ Lastly, HMGA1 regulates the symmetric/asymmetric cell division ratio and self‐renewal in CSCs through transcriptional regulation of p53.^72^ However, HMGA1‐relevant small molecule compounds or drugs are rarely reported. Only one paper suggests that HMGA1 proteins led to chemoresistance against drugs such as cetuximab and 5‐fluorouracil.^73^

In hepatobiliary cancer, there are few relevant studies. These studies were mainly focused on HMGA1 expression and correlated with worse clinical outcome.^32^, ^74^ HMGA1 has negative expression in hepatocellular carcinoma and different degrees of positive expression in intrahepatic cholangiocarcinoma and metastatic adenocarcinoma to the liver, especially in the metastatic lesions from pancreatic carcinoma, where it shows 100% positive expression.^75^ Another paper shows that HMGA1, a predictive marker in hepatocellular carcinoma, is involved intrahepatic metastasis, rather than non‐intrahepatic metastasis.^76^ There is only one paper on cholangiocarcinoma. Studies show that HMGA1 enhances tumourigenicity and confers resistance to therapy. This paper indicates that HMGA1 promotes colony formation, cell proliferation and resistance to gemcitabine treatment.^77^

The quantitative immunohistochemical analysis in HMGA1 transgenic mice aged 5, 11 and 15 months showed that HMGA1 expression is significantly increased in pancreatic intraepithelial neoplasia.^78^ HMGA1 serves as a potential diagnostic molecular marker in intraductal papillary mucinous tumours and pancreatic duct cell carcinomas.^79^ A series of findings may have determined the molecular function of HMGA1. At the transcriptional level, HMGA1 transcription induces the expression of cyclooxygenase 2 (COX‐2) or regulates the insulin receptor to increase cyclin D1 translation and later promotes malignant progression.^80^, ^81^ The roles of the phosphatidylinositol 3‐kinase (PI3‐K)/Akt signalling pathway are very important in HMGA1 inducing pancreatic carcinoma. Relevant studies note that HMGA1 led to cellular invasiveness and metastasis in a PI3‐K/Akt‐dependent mechanism through Akt phosphorylation at Ser (473).^82^ Based on the preclinical models of drug resistance, chemosensitivity is increased when HMGA1 expression is suppressed,^83^ and HMGA1 promotes the chemoresistance of pancreatic carcinoma to gemcitabine.^84^ Studies indicate that the PI3‐K/Akt pathway has an important role in the specifics of HMGA1 mediated chemoresistance.^85^ Li et al report that metformin up‐regulated the expression of miRNA, such as let‐7c, miR‐26a and miR‐192, in a dose‐dependent manner. These miRNAs can inhibit pancreatic cancer proliferation. One of these miRNAs, miR‐26a directly targets HMGA1 to inhibit pancreatic cancer progression. Therefore, we can conclude that metformin has a role in the treatment pancreatic cancer.^86^

## 6 REPRODUCTIVE SYSTEM CANCER

**Figure 4:**
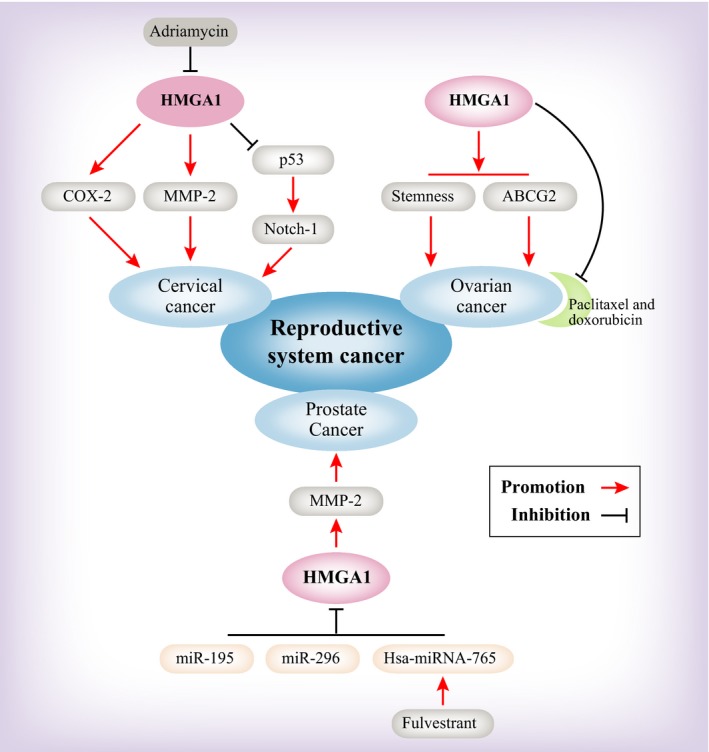
A model depicting the upstream and downstream regulation of HMGA1 in reproductive system cancers

There are many papers that reveal that HMGA1 induces malignant progression and serves as a potential clinical biomarker in reproductive system cancer. PCa is the most investigated cancer in the male genitourinary system, while in the female reproductive system, ovarian carcinoma, uterine cancer, cervical cancer and endometrial cancer studies have been reported in recent decades. We review these studies individually (Figure 4).

In PCa, HMGA1 has been shown to be associated with the nuclear matrix. In examining the HMGA1 protein level during the progression from normal to prostate neoplasia in HMGA1 transgenic mice, the data show that HMGA1 expression is correlated with malignant properties in PCa.^34^ Because the prostate belongs to the male internal genital organ and it can be influenced by androgen, HMGA1 could transform PCa cells from androgen‐sensitive to androgen‐insensitive and could play a vital role in the cell proliferation of androgen‐independent PCa cells.^87^ It is well known that HMGA1 is highly expressed in embryogenesis, as well as in most malignant neoplasms, and it has been found in post‐pubertal testicular germ cell tumours, including in seminomas and embryonal carcinomas. Chieffi et al reported that HMGA1 has high expression in testicular seminomas.^88^ They also revealed that HMGA1 interacts with estrogen receptor β (ERβ) in nuclear of germ cells; however, such interaction is impaired by the absence of ERβ in testicular seminomas.^89^ Additionally, HMGA1 represents a valid diagnostic marker by immunohistochemistry analysis. How HMGA1 performs its function is subsequently reviewed. The miRNA‐microarray analysis in PCa patients has identified several candidate miRNAs (let‐7 family, miR‐181b, ‐515‐3p/5p, ‐361 and ‐146b) with differential expression, especially let‐7b, which targets HMGA1 to inhibit PCa.^90^ Other miRNAs, including miR‐195 and miR‐296, perform a similar function to inhibit the expression of HMGA1.^91^, ^92^ Importantly, fulvestrant treats a novel PCa pathway through ERβ‐mediated transcriptional up‐regulation of hsa‐miR‐765.

Studies note that HMGA1 serves as a useful diagnostic biomarker in ovarian carcinomas. HMGA1 has high expression in malignant cancer, as determined through immunohistochemistry, when comparing normal tissue to malignant ovarian cancer.^93^ Liu et al indicates that using short/small hairpin RNAs of HMGA1 led to decreased growth and metastasis potential of ovarian cancer.^94^ In a non‐invasive urinary detection test, HMGA1 is correlated with the degree of malignancy of ovarian cancer. It has been verified that HMGA1 served as a non‐invasive biomarker in ovarian diagnosis.^35^ How HMGA1 performs its function in ovarian cancer is subsequently reviewed. Studies indicated that overexpression of HMGA1 can elevate Spheroid‐forming cancer stem cells, increasing stemness‐related gene expression, such as ALDH, SOX2, ABCB1, ABCG2 and KLF4. At the same time, ovarian cancer showed resistance to chemotherapeutic agents, such as doxorubicin and paclitaxel.^95^

Studies show that HMGA1 is an oncoprotein in cervical cancer; however, the molecular underpinnings of malignant progression remain poorly understood. Human papilloma virus (HPV) infection is an important factor in cervical cancer. Studies have shown that HMGA1 expression is sustained by HPV E6/E7 proteins, and a positive autoregulatory loop has been established between these proteins in cervical cancer.^96^ By generating transgenic mice with HMGA1a, the following research shows that the HMGA1a protein level is increased in high‐grade cancer but not in normal uterine tissue. Additionally, these studies found that HMGA1a binds directly to the COX‐2 promoter based on chromatin immunoprecipitation.^97^ At the same time, Di Cello et al also demonstrated that COX‐2 inhibitors can rescue the role of HMGA1 in cervical cancer.^98^ Cervical cancer growth is impaired in an matrix metalloproteinase‐2 (MMP‐2) deficient background in HMGA1a transgenic mice. This finding indicated that HMGA1 positively regulated MMP‐2 to induce cervical cancer.^11^

## 7 CONCLUSIONS

HMGA1 is an oncofoetal protein that participates in tumourigenesis and tumour progression. As an oncogene, HMGA1 is up‐regulated in many different tumours, including epithelial and mesenchymal tissue‐originated tumours, as shown in this review. HMGA1 overexpression is correlated with poor clinical outcome, distant metastasis and advanced tumour stage in many cancers. As an architectural transcription factor, HMGA1 is involved in many biological pathways, such as the TNF‐α/NF‐κB pathway, EGFR pathway, Hippo pathway, Ras/ERK pathway, Akt pathway, Wnt/beta‐catenin pathway and PI3‐K/Akt pathway. In these biological pathways, HMGA1 targets different downstream genes. However, most of the studies on HMGA1 upstream genes are miRNAs. Most of the studies reported that miRNAs, such as miR‐15, miR‐16, miR‐26, miR‐214, miR‐296, miR‐761 and Let‐7a, can inhibit the expression of HMGA1, thereby suppressing tumour progression. Additionally, Zhang et al reported that HMGA1 can promote the development of NSCLC by promoting miR‐222 expression. There is emerging evidence that HMGA1 is a vital tumourigenic factor in the tumours of every functional system, but most of the relevant studies are in breast cancer and colorectal cancer. HMGA1 as an oncogene has been able to provide certain evidence for clinical diagnosis. Further studies of the targets of the HMGA1 gene, such as small molecules or drug interventions, will be useful for the clinical treatment of tumours. It has been reported that HMGA1 leads to tumour resistance to chemotherapeutic drugs by maintaining the stemness property of the tumour cells. Akhter et al found that Adriamycin can interact with HMGA1 and is involved in the inhibition of HMGA1 and its role in cervical cancer. Other studies on small molecules or drugs targeting HMGA1 are few, and more studies are needed for further discovery.

## DISCLOSURE

No potential conflicts of interest were disclosed.

## AUTHORS’ CONTRIBUTION

YW, LG conceptualized and designed the study; also written, reviewed and revised the manuscript. YW, LH developed the methodology. YW, LH, YZ analysed and interpreted the data. LG supervised the study.
